# A Systematic Review and Meta-Analysis on the Effectiveness of Interventions in Reducing Missed Opportunities for Vaccination Among Children Under Age Five

**DOI:** 10.3390/vaccines14060505

**Published:** 2026-06-04

**Authors:** Jacques L. Tamuzi, Patrick D. M. C. Katoto, Doris Y. Sakala, Serge M. Zigabe, Charles S. Wiysonge, Peter S. Nyasulu

**Affiliations:** 1 Division of Epidemiology and Biostatistics, Department of Global Health, Faculty of Medicine and Health Sciences, Stellenbosch University, Cape Town 7505, South Africa28771907@sun.ac.za (D.Y.S.); shey.wiysonge@mrc.ac.za (C.S.W.);; 2 Cochrane South Africa, South African Medical Research Council, Cape Town 7505, South Africa; 3 Centre for Tropical Diseases and Global Health, Department of Medicine, Catholic University of Bukavu, Bukavu 285, Democratic Republic of the Congo; serge.zigabe@kuleuven.be; 4 General Pediatrics, Pediatric Department, Hôpital Provincial Général de Référence de Bukavu, Bukavu 285, Democratic Republic of the Congo; 5 Centre for Environment and Health, Department of Public Health and Primary Care, KU Leuven, Herestraat 49, 3000 Leuven, Belgium; 6 Division of Epidemiology and Biostatistics, School of Public Health, Faculty of Medicine and Health Sciences, University of the Witwatersrand, Johannesburg 2050, South Africa

**Keywords:** MOVs, under-five children, interventions, meta-analysis, systematic review

## Abstract

Introduction: A knowledge gap exists around which interventions can be implemented to minimise missed opportunities for vaccination (MOVs) in children under age five. Children under age five experience higher rates of MOVs, making them a critical focus for interventions to reduce MOV. This study aims to plot evidence-based interventions reducing MOVs in children under age five. Methods: We performed an electronic search without any language restriction for studies indexed between 1 January 1990 and 30 August 2025 in PubMed (Medline), Web of Science, Cochrane Central Register of Controlled Trials, Scopus, CINAHL, and other sources. We included studies highlighting interventions reducing MOVs in children under age five. A meta-analysis was conducted using the random-effects model with risk ratios (RRs) and 95% confidence intervals (CIs). When meta-analysis was not possible, results were reported individually. Results: Out of 535 papers reviewed via titles and abstracts, 21 were included in the meta-analysis. Overall, education interventions reduced the risk of MOVs by 35% (RR 0.65, 95% CI 0.47 to 0.91). Our results also showed that electronic immunisation registries (EIRs) and alerts were successful in lowering the risk of MOVs by 29% (RR 0.71, 95% CI 0.54 to 0.92). Integrated delivery of health interventions reduced the risk of MOVs by 66% (RR 0.34, 95% CI 0.14 to 0.83). Other interventions that reduced the risk of MOVs included integrating a pharmacist in the under-five vaccination programme (RR 0.43, 95% CI 0.26 to 0.72) and multicomponent interventions (RR 0.62, 95% CI 0.48 to 0.80). Financial incentives (RR 0.87, 95% CI: 0.80, 0.94) and combined vaccines (RR 0.72, 95% CI 0.68 to 0.76) showed a statistically significant MOV reduction. However, our results were graded from very low-to-moderate grade evidence. Conclusions: Education interventions, EIRs and alerts, pharmacist inclusion in vaccination programme, integrated delivery of health interventions, multicomponent interventions, combined vaccines, and financial incentives may improve MOVs in children under age five as the evidence was graded from very low to moderate.

## 1. Introduction

Vaccinations are considered cost-efficient public health interventions, and have been instrumental in the reduction and impact of several vaccine-preventable diseases (VPDs) [1,2,3]. The revised WHO technique for assessing missed opportunities for vaccination (MOVs) was endorsed by the Strategic Advisory Group of Experts on Immunisation (SAGE) in 2016 [4]. Among the standardised methodology of three phases of MOV assessment, the intervention phase should be viewed as substantial, similar to targeting MOV barriers and decreasing MOVs in a target population [4]. The study defines a MOV as a child who is eligible for vaccination, has no contraindications, but is not receiving all recommended doses of vaccinations during a health care visit [5,6]. In 1993, a systematic review including 45 countries found an estimated MOV prevalence of 67% [7]. More than three decades later, MOVs remain one of the most important factors lowering vaccination coverage in resource-limited settings, with a prevalence of 34% (24–43%) reported in a systematic review conducted in 2025 [8]. This demonstrates relatively minor gains in reducing MOVs in the past thirty years [8]. Thus, effective interventions to address MOVs may help governments meet immunisation targets and improve the timeliness of vaccine delivery. Approaches include notifying communities about forthcoming vaccinations, recalling them when a dose is missed, and directly tackling MOVs may help to increase vaccination rates and reduce the burden of vaccine preventable diseases (VPDs) [3]. Research shows that frequent evaluation of immunisation systems, identification of barriers to new vaccine uptake and implementing context-specific interventions are critical to minimising MOVs, and thereby increasing vaccine coverage [8,9].

Evidence suggests that the burden of MOVs is especially significant among children under age five in low-and-middle income countries [10], where are the major target group of routine immunisation programme. In fact, the socioeconomic processes impacting under-five vaccination are notably different to those affecting teenage or adult immunisation as those mechanisms broadly depend on the caregivers. A significant proportion of children under age five who visit health facilities for other services leave without receiving the necessary vaccines, highlighting gaps in health-system practices such as inadequate vaccination screening and missed integration of immunisation with other child health services [7,11]. An estimated 15% (6–24%) of MOVs remain uncorrected among children under age five in resource-limited countries [8]. Knowing that VPDs tend to cause the highest mortality and morbidity in children under 5 years of age, these uncorrected MOVs may represent an important and preventable source of the continued burden of VPDs. Addressing MOVs among children under age five is thus critical for improving vaccination coverage and reducing the burden of VPDs in this vulnerable population [7,11,12]. Recent systematic reviews and meta-analyses highlighting how interventions may minimise MOVs in children under age five are lacking. Most of the existing systematic reviews related to MOVs have mixed different age ranges without any specification [1,5,13]. Though, none of them have included a meta-analysis to estimate the overall plots of MOV interventions without any specification in children under age five. Thus, a knowledge gap exists around which interventions can be effectively implemented to minimise MOVs in children under age five. However, implementing MOV interventions is integral to the WHO approach for minimising MOVs [14]. Then, conducting a systematic review and meta-analysis in children under age five is substantial as this age range experiences higher rates of MOVs than other infants [9,15], making the children under five age group a critical focus for interventions to reduce MOVs. This study aims to plot evidence-based interventions for reducing MOVs in children under age five.

## 2. Methods

A PICO search framework was established with the following components: Population (Caregivers of children under age five eligible for vaccinations, health workers involved in immunisation programme, and any individuals involved in children under age five immunisation), Intervention (any interventions implemented to reduce MOVs), Comparison (standard care, routine immunisation services, no intervention, or other alternative interventions), and Outcome (rate of MOVs in children under age five). This systematic review and meta-analysis was reported according to the PRISMA (Preferred Reporting Items for Systematic reviews and Meta-Analyses) guidelines [16]. This systematic review was registered in the International prospective register of systematic reviews (PROSPERO) [(https://www.crd.york.ac.uk/PROSPERO/view/CRD420251155509 (accessed on 29 September 2025)]. The comparison of the final systematic review and the PROSPERO record verified full consistency in objectives, eligibility criteria, search technique, outcome definitions, data synthesis and planned analyses.

### 2.1. Electronic Search Data

A search of the literature was systematically conducted in August–September 2025 using PubMed (Medline), Scopus, Web of Science, Cumulative Index to Nursing and Allied Health Literature (CINAHL), the Cochrane Central Register of Controlled Trials (CENTRAL), and Google Scholar. Further searches were conducted in the grey literature (WHO and GAVI, Alliance websites). The search was conducted without any language restriction for studies published between 1 January 1990 and 30 August 2025. The full search strategy was described in the Supplemental Material S1. Studies found in other languages were translated in English using Google translate. All retrieved records were imported into Covidence [(https://www.covidence.org/ (accessed on 17 July 2025)], where duplicate citations were automatically identified and removed before title and abstract screening.

### 2.2. Inclusion and Exclusion Criteria

All studies including interventions to reduce MOVs for children under age five in any country were included. Included interventions were those targeting caregivers, health providers, health systems, and any combination of the above categories of interventions compared to standard care or routine vaccination services, no intervention, pre-intervention vs. post-intervention comparisons or other alternative interventions. Only randomised control trials, cluster randomised trials, quasi-experimental, controlled before-and-after, and cohort studies were included. Only studies that reported at least MOVs as the outcome were included. Eligible studies where full texts could not be obtained were excluded. Furthermore, studies including all children, without any possibility of separating those under age five, were excluded as well.

### 2.3. Interventions

All potential interventions reducing MOVs in children under age five were included. The following interventions were included: education, short message service (SMS), phone, electronic records and reminders, pharmacist inclusion in vaccination program, outreach, post cards, financial incentives, free vaccination programme, integrated management of childhood illness (IMCI), and combined vaccines. Education interventions referred to any intervention consisting of educating the healthcare providers regarding this policy and the need to assess vaccination status at each visit. In this review, education interventions were reviewed based on interactive sessions including healthcare providers about the importance of childhood vaccination, the limited contraindications and the role of hospital visits for MOVs. Education interventions also consist of educating caregivers about understanding the vaccination cards or records, immunisation timing, safety, and contraindications. After caregivers’ knowledge assessment, further education interventions focused on perceived barriers by addressing false contraindications to immunisation and self-efficacy and by empowering caregivers to take an active role in their child’s health care.

SMS and phone reminders and electronic immunisation registers (EIRs) with alerts and scheduled vaccination interventions included case managers who followed up with clients by telephone and mail after scheduled vaccination appointments for their children under age five. Moreover, the system generated alerts for all clinicians involved in either office visits or telephone-based care. An especially interesting approach for improving MOVs in paediatric settings involves pairing EIRs with alerts provided to clinicians during patient care [17,18]. IMCI’s objective is to reduce mortality, morbidity and disability, while contributing to improved growth and development status among infants and children under five age [19]. IMCI studies include both preventive and therapeutic interventions that are implemented by health workers in facilities with the aim of improving vaccination uptake in children aged less than 5 years. Eventually, the intervention clinic was staffed by a board-certified ambulatory clinical pharmacist who was a full-time part of the health care team [20,21]. The pharmacist took advantage of each patient’s visit to the clinic to screen their medical record for eligibility for MOVs [20]. In the same line, the pharmacist developed a personalised immunisation plan for eligible patients and communicated that plan to the provider, either verbally or by documentation in the EIRs [20]. The pharmacist did this, in addition to their regular tasks, which included counselling patients, handling insurance difficulties, completing medication reconciliation, and recommending relevant treatments [20]. Finally, the inclusion of combined vaccines has simplified their introduction into recommended vaccination schedules by lowering the number of injections required. Consequently, this has improved immunisation compliance and reduced MOVs during single clinic visits. In addition, other interventions, such as multicomponent interventions, free immunisation programme, vaccination services located close to consulting room, physician recommendations for vaccination, and financial sanctions, may play a substantial role in reducing MOVs. Interventions to reduce MOVs in children under age five were classified as recipient- and provider-oriented, health system-oriented, or multicomponent interventions, as indicated by Oyo-Ita et al. [11]. This intervention classification was most suitable for this systematic review because of its diverse classification based on caregivers, health system access, and provider practices, as recommended by the WHO.

### 2.4. Outcome Definition

The main emphasis of this review was MOVs among children less than age five. Both preventive treatments and acute care visits may create instances when MOVs can occur [5,6]. MOVs serve as a surrogate indicator for vaccination coverage, as a reduction in MOVs corresponds to an increase in vaccination coverage [5].

### 2.5. Data Extraction and Synthesis

A data extraction form was designed to collect information regarding the study ID, country, objectives, population age, study design, type of interventions, interventions’ duration, and the outcome. Three authors screened the titles and abstracts to find potential studies. Any discrepancy was solved by consensus. Data extraction was completed by three reviewers (J.L.T., S.M.Z., and D.Y.S.) and any discrepancies resolved through discussion or by consulting a third author (P.S.N.).

A meta-analysis was undertaken to plot interventions reducing MOVs in children under age five. Results were plotted based on intervention similarities, including education, EIRs and alerts, phone calls and SMS, integrated delivery of health interventions, presence of a pharmacist in immunisation service, and multicomponent interventions. Meta-analysis via the Mantel–Haenszel method including random effects was used to pull the RRs and their 95% CIs. Heterogeneity was quantified using the ꭕ^2^ test and *I*^2^. When the *I*^2^ was computed at 50% and above, this represented high heterogeneity. In case of high heterogeneity, a subgroup analysis was conducted comparing intervention subsets. Leave-one-out sensitivity analysis was done to investigate the influence of each study on the overall effect-size estimate and to identify influential studies for each intervention group. R version 4.5.2 with R Studio version 2025.9.2 was used to undertake the meta-analysis. Further analysis, including risk of bias assessment graphs and systematic assessment of evidence certainty, were performed using Revman software 3.5, and GradePro version 3.6.1, respectively. In instances when meta-analysis was impracticable, studies were presented individually.

### 2.6. Missing Data

Authors were solicited to provide missed data. We used software to digitise and extract data from graphs when numerical values are not provided. Finally, we documented the total number of individuals randomised in some studies, as the number analysed in each group was not provided in several publications, and the authors did not respond to our enquiries.

### 2.7. Risk of Bias Assessment

Three reviewers (J.L.T., S.M.Z., and D.Y.S.) were used to assess the risk of bias and any divergence was solved by consensus or by consulting a third author (P.S.N.). For randomised controlled trials (RCTs), the Cochrane risk-of-bias tool was used to assess quality. The following domains were assessed: selection, allocation concealment, performance, detection, attrition, reporting and other bias [22]. Each study was judged as having high, low, or unclear risk of bias for each domain. For non-randomised studies of interventions, our analysis applied the criteria established by the Cochrane Effective Practice and Organization of Care group [22]. Additional considerations to assess study bias as high, low or uncertain were baseline characteristics, outcomes, contamination and selective reporting. Subsequently, we summarised the overall evidence quality according to GRADE criteria [23], assigning a final grade of high, moderate, low, or very low [24]. Grading the effects of interventions was assessed by using the limitations in the design or execution of randomised trials, inconsistency, indirectness, imprecision, and dissemination bias [25].

## 3. Results

### 3.1. Description of Included Studies

**Figure 1:**
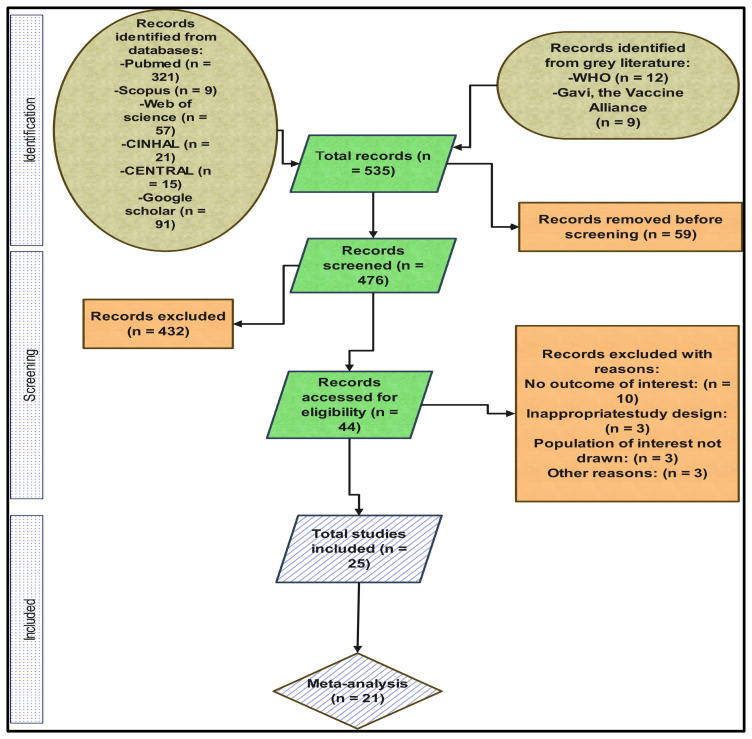
Prisma Flow diagram of interventional studies targeting MOVs in children under age five.

Out of 535 records, 59 were duplicates, and 476 were screened through titles and abstracts. Of these, 433 were excluded as they were irrelevant, while 43 records were assessed as full texts and 21 studies were included in the meta-analysis, with a further four studies reported individually (Figure 1). Twenty-five studies that assessed MOV interventions in children under age five were included [18,20,21,26,27,28,29,30,31,32,33,34,35,36,37,38,39,40,41,42,43,44,45,46,47]. Of these, fifteen studies were conducted in the USA [18,20,21,27,28,30,31,32,33,35,38,39,43], two studies in Nigeria [36,37] and one study in each of the following countries: South Africa, India, Pakistan, Bangladesh, Côte d’Ivoire, Tanzania, and South Sudan [26,29,34,40,41,47]. Regarding the study designs, seven studies were RCTs [18,27,31,33,35,42,43] and the other studies were quasi-experimental and cohort studies [20,21,26,28,29,30,32,34,36,37,47]. Among studies reported individually, one study was from Sudan [48] and three studies were from the USA [28,38,39]. In terms of the study’s population, eight studies included children under two years [18,29,31,34,35,37,39,42], four studies included children under age five [20,21,26,47], two studies included children aged 12–23 months [28,49], two studies included children aged 2 to 5 years [34,43], four studies included children aged less than 9 months [30,33,36,48], and four studies included children under three years [27,32,38,50]. The characteristics of the included studies are described in Appendix A and the list of excluded studies is provided in Supplemental Material S2.

### 3.2. Risk of Bias Assessment

**Figure 2:**
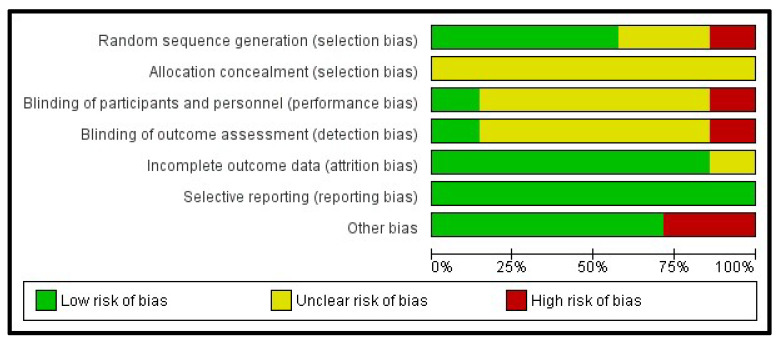
Risk of bias assessment of randomised control trials.

In total, seven RCTs and thirteen quasi-experimental studies included in the meta-analysis were assessed for the risk of bias. Four of the RCTs used random sequence generation. Similarly, none of the randomised control studies included allocation concealment. Only one RCT included participant and staff blinding, or outcome assessment blinding (Figure 2). In 80% of the RCTs, inadequate outcome data was reduced, and all RCTs had no reporting bias. Other types of bias were detected in two RCTs (Figure 2).

**Figure 3:**
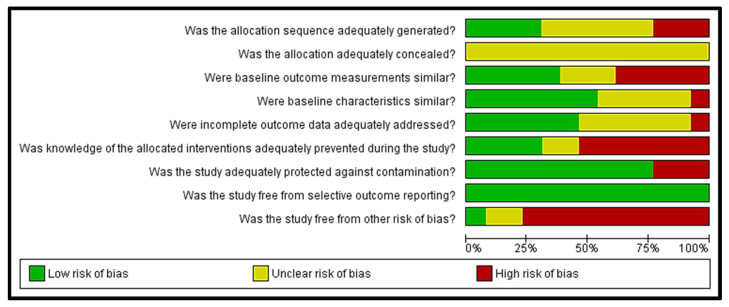
Risk of bias assessment of quasi-experimental studies.

Only 31% of the quasi-experimental studies indicated that the allocation sequence was appropriately generated. None of them were found to have the allocation adequately concealed. In 38% of the quasi-experimental studies, the baseline result measures were identical, as were the baseline characteristics in 54% of trials (Figure 3). Inadequate outcome data was managed properly in 46% of studies. In 31% of the quasi-experimental studies, knowledge of the allocated interventions was adequately prevented during the study (Figure 3). In the same line, 77% of the quasi-experimental studies were adequately protected against contamination. Additionally, all studies were found free from selective outcome reporting and 8% of the studies were found free of other risks of bias (Figure 3).

### 3.3. Effects of Interventions

#### 3.3.1. Recipient- and Provider-Oriented Interventions

##### Education Interventions

**Figure 4:**
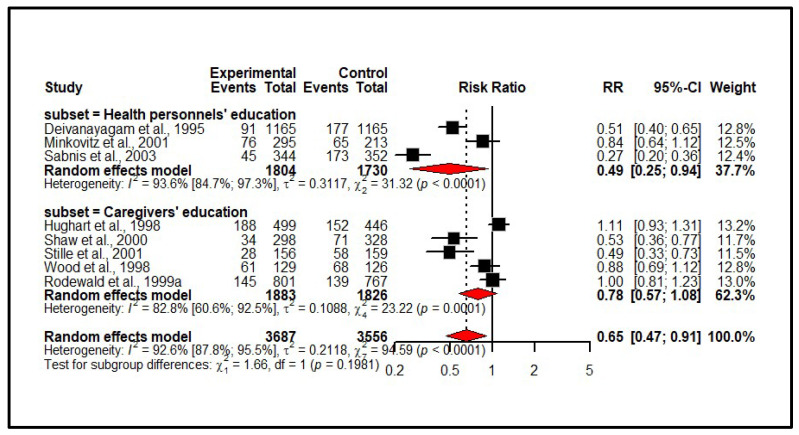
Effectiveness of education intervention reducing MOVs in children under age five [27,29,30,31,32,33,42,44].

Our findings indicated that health personnels’ education was more beneficial in lowering MOVs (RR 0.49, 95% CI 0.25 to 0.94, three studies, 3534 children under age five) than caregivers’ education (RR 0.78, 95% CI 0.57 to 1.08, five studies, 3709 children under age five) (Figure 4). Overall, educational interventions reduced MOV risk by 35% (RR 0.65, 95% CI 0.47 to 0.91, eight studies, 7.243 children under age five). The heterogeneity of the studies was substantial, with *I*^2^ = 93.6% (95% CI: 84.7–97.3%) for health personnels’ education and *I*^2^ = 82.8% (60.6–92.5%) for caregivers’ education (Figure 4). The overall heterogeneity was estimated at 92.6% (95% CI: 87.8–95.5%). However, the test for subgroup differences was not statistically significant (*p*-value = 0.20) (Figure 4). An assessment of the grading approach found that education received very low evidence ratings. Omitting Sabnis et al., 2003 [32], the overall plot revealed the lowest MOV reduction related to education intervention, with a 25% reduction (RR 0.75, 95% CI 0.58 to 0.96) and when Hughart et al., 1998 [30] was omitted, the overall plot revealed the highest MOV reduction, estimated at a 40% reduction (RR 0.60, 95% CI 0.46 to 0.96) (Supplemental Figure S1).

##### Electronic Immunisation Registers and Alerts Interventions

**Figure 5:**
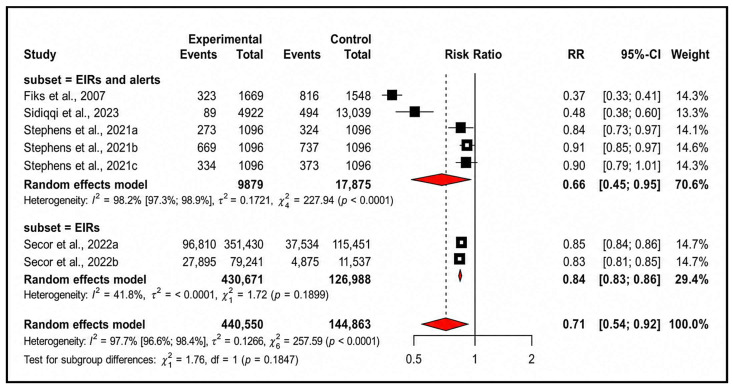
Effectiveness of EIRs and alerts interventions in reducing MOVs in children under age five [18,41,43,47].

EIRs and alerts showed a 29% risk reduction in MOVs (RR 0.71, 95% CI 0.54 to 0.92, four studies, 585,413 children under age five) (Figure 5). Heterogeneity across studies including EIRs and alerts was substantial, with *I*^2^ = 97.7% (95% CI: 96.6–98.4%). However, the test for subgroup differences was not statistically significant (*p*-value = 0.185). In comparison to EIRs (RR 0.84, 95% CI: 0.83–0.86), EIRs and alerts showed a MOV reduction of 34% (RR 0.66, 95% CI: 0.45–0.95) (Figure 5). Knowing that both high-income and resource-limited countries were included in this analysis, the subgroup analysis did not show any statistically significant difference between the two settings (*p* = 0.995) (Supplemental Figure S2). Based on EIRs’ evolving landscape of immunisation, the single study of EIRs completed in 2007 (Fiks et al., 2007) [18] revealed statistically significant differences from those conducted after 2020 (Supplemental Figure S3). EIRs and alerts were rated as low on the evidence scale. In the leave-one-out sensitivity analysis, omitting Fiks et al., 2007 [18] showed the highest pooled results of EIRs and alerts of RR 0.80, 95% CI 0.67 to 0.97, and omitting Siddiqi et al., 2023 [41] revealed the pooled estimate of RR 0.75, 95% CI 0.57 to 0.93 (Supplemental Figure S4).

##### SMS and Phone Call Reminders Interventions

**Figure 6:**
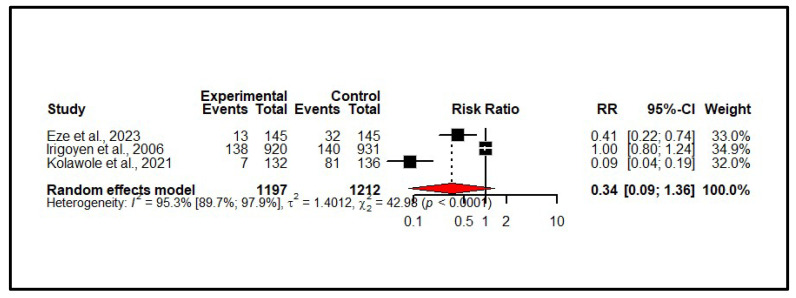
Effectiveness of SMS and phone call reminder interventions in reducing MOVs in children under age five [36,37,45].

Our results demonstrated that SMS and call reminders were successful in lowering MOVs in under-five children, with a 64% risk reduction (RR 0.34, 95% CI: 0.09 to 1.36, three studies, 2409 children under age five). However, the SMS and call reminders overall result was not statistically significant (Figure 6). High heterogeneity was found in studies including SMS and calls reminders, with *I*^2^ = 95.3% (89.7–97.9%) (Figure 6). An assessment of the grading approach found that SMS and phone reminders received very low evidence ratings. The leave-one-out sensitivity analysis showed that when omitting Irigoyen et al., 2006 [45], the estimated plot was statistically significant with RR 0.19, 95% CI 0.04 to 0.90 (Supplemental Figure S5).

#### 3.3.2. Health System Interventions

##### Integration of Vaccination Services with Other Services

**Figure 7:**
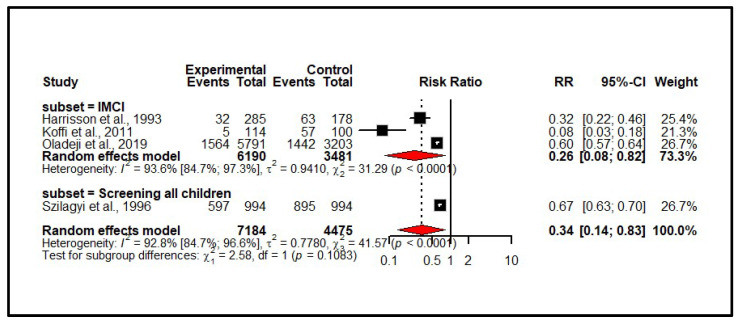
Effectiveness of integrated delivery of health interventions in reducing MOVs in children under age five [26,34,35,40].

Integrated delivery of health interventions reduced MOV risk in children under age five by 66% (RR 0.34, 95% CI 0.14 to 0.83, four studies, 11,659 children under age five) (Figure 7). Integrated delivery of health was classified in two groups, mainly IMCI (RR 0.26, 95% CI 0.08 to 0.82, three studies, 9671 children under age five) and screening all children (RR 0.67, 95% CI 0.63 to 0.70, one study, 1988 children under age five) (Figure 7). There was significant heterogeneity across studies, with *I*^2^ = 93.4% (95% CI: 84.7–96.6%) (Figure 7). Furthermore, the test for subgroup differences was statistically significant (*p* = 0.04). An assessment of the grading approach found that IMCI interventions were rated as very low on the evidence scale. Omitting Szilagyi et al., 1996 [35], the overall integrated delivery of health interventions was estimated at RR 0.28, 95% CI 0.12 to 0.64 and omitting Koffi et al., 2011 [40], the pooled result was estimated at RR 0.58, 95% CI 0.49 to 0.68 (Supplemental Figure S6).

##### Pharmacist Integration in Vaccination Programme

**Figure 8:**
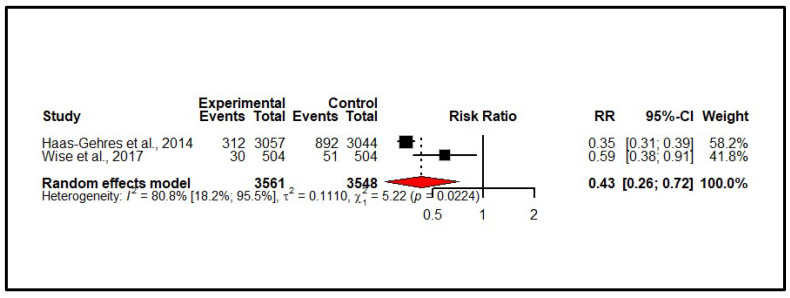
Effectiveness of pharmacist integration in under-five vaccination programme in reducing MOVs in children under age five [20,21].

Our findings also showed that pharmacist inclusion in the under-five vaccination programme had a MOV risk reduction of 57% (RR 0.43, 95% CI 0.26 to 0.72, two studies, 7109 children) (Figure 8). However, the heterogeneity across the two studies was moderate, with *I*^2^ = 80.8% (95% CI: 18.2–95.5%) (Figure 8). An assessment of the grading approach to pharmacist integration in the under-five vaccination programme was rated as low on the evidence scale.

##### Other Health System Interventions

A pre-intervention and post-intervention study design investigating the effect of free vaccination programmes on MOVs did not show any difference (RR 1.00, 95% CI: 0.95, 1.05) (low-grade evidence) [38]. A randomised cross-over study comparing when the place for vaccination was moved very close to the consulting room vs. when the doctor seeing the infant wrote a prescription recommending vaccination for the child did not show effective MOV reductions between the two interventions (RR 2%; 95% Cl −4% to +7%) (low-grade evidence) [48]. In the same line, an RCT assessing financial incentives vs. control groups showed a significant risk reduction in MOVs of 13% (RR 0.87, 95% CI: 0.80, 0.94) (moderate-grade evidence) [39]. Moreover, a quasi-experimental study comparing combined vs. monovalent vaccines administration showed a statistically significant MOV reduction (RR 0.72, 95% CI 0.68 to 0.76) (low-grade evidence) [28].

#### 3.3.3. Multicomponent Interventions

**Figure 9:**
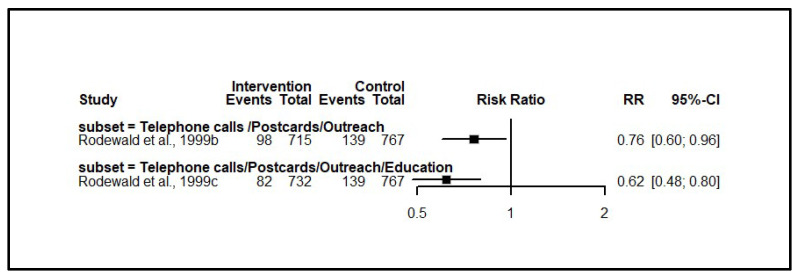
Effectiveness of multicomponent interventions in reducing MOVs in children under age five [42].

Only one study used multicomponent interventions, with two distinct interventions compared to one control group. Multicomponent interventions including telephone calls, post cards, outreach, and caregivers’ education showed a 38% MOV reduction (RR 0.62, 95% CI 0.48 to 0.80, 1499 children under age five) compared to the combinations of telephone calls, post cards, and outreach (RR 0.76, 95% CI 0.60 to 0.96, 1482 children under age five). The heterogeneity was estimated at 22.8% (Figure 9). An evaluation of the grading approach for multicomponent interventions was rated moderate.

## 4. Discussion

This systematic review was designed to look at evidence-based interventions decreasing MOVs in children under age five. Overall, our results showed that health personnel and caregivers’ education EIRs and alerts, integrated delivery of health interventions, pharmacist integration in vaccination programme, multicomponent interventions, combined vaccines, and financial incentives were effective in reducing MOVs in children under age five. Our results were in the line with previous systematic reviews assessing MOV interventions [1,5,13]. Several studies indicated that children born to mothers who had no formal education had a higher risk of MOVs than those born to mothers who had completed secondary school or higher [9,51]. We suggest that education interventions may play a substantial role in understanding the vaccination cards or records, vaccine hesitancy, immunisation timing, safety, and false contraindications. Furthermore, education interventions could boost knowledge of healthcare providers about the importance of childhood vaccination, the limited contraindications and the role of hospital visits for MOVs. Additionally, educational interventions for use in settings with high numbers of uneducated caregivers like sub-Saharan Africa may be potential interventions especially for a long-term MOV reduction. EIRs, especially when coupled with point of care alerts to clinicians, are an efficient means of reducing MOVs. These systems have been shown to be reliable and competent to identify children with immunisation delays [18,45]. A standardised immunisation schedule is required for the development of EIRs and a related alarm system. An EIR-based strategy coupled with alerts may constitute important interventions in reducing MOVs in resource-limited countries. This was supported by our findings, which show that EIRs and alerts reduced MOVs more effectively than EIRs alone. In the same way, the combination of patient reminders or recall with provider reminder systems was the most effective intervention to improve immunisation rates according to a systematic review and meta-analysis [3]. Additionally digital applications or platforms for EIRs such as web-based and mobile-based applications, can potentially improve childhood vaccination services by automatically updating the child’s immunisation schedule after every visit, adjusting for missed appointments and delayed vaccinations as documented in Kenya, Benin, Burkina Faso, Nigeria, Ghana, Tanzania, Zimbabwe, Pakistan, and Bangladesh [41,47,52]. This example shows that artificial intelligence (AI)-powered EIRs in under-five immunisation may enhance worker capacity, optimise vaccine supply, and expand access to health information and personalised reminders about upcoming vaccination doses, ensuring adherence to schedules leading to more effective and widespread vaccination coverage in the future [53] and a substantial lead in MOV reduction.

Integrated delivery of health interventions has shown to exploit MOVs far more effectively than one in which preventive and curative services are rendered separately [26,34]. In addition to the fact that integrated delivery of health interventions ensures that far fewer opportunities for immunisation are missed, another important benefit is that children who have been missed by the system and have not completed their standard primary immunisations will be recognised when they show up as sick patients [34]. A policy aimed at minimising MOVs would improve mandatory screening for immunisation status in all children under age five. A study conducted in Uganda and Nepal demonstrated that mandatory screening for immunisation status in children under age five elevated vaccination coverage from 75% to 96% and from 50% to 91%, respectively [54,55]. This is particularly important in populations with high under-five mobility, as is the case in most resource-limited countries. Pharmacists embedded in primary care could improve paediatric vaccination uptake [20]. Pharmacists are medicine experts, familiar with the regular and catch-up immunisation schedules, and able to identify those at higher risk. They are also equipped to conduct systematic vaccination status screening, defaulter tracing and follow-up, relevant contraindications to vaccines, vaccine stock management, and cold chain maintenance [20,56]. Pharmacists can use relationships with families to highlight the risks of not vaccinating children and dispel misinformation about vaccinations. Pharmacists also consult with clinicians to recommend the right immunisations based on patient medical histories and review providers’ final vaccine orders [20]. The recommendation of combined vaccines administration is also considered to be an effective strategy for improving the vaccination coverage, by minimising MOVs in children under age five. This might be explained by the fact that the availability of a combination vaccination would make doctors more inclined to suggest, or parents more likely to accept, all the required shots at any one appointment [28], as combining numerous antigens into a single vaccine minimises the number of doses a child needs, making the immunisation procedure easier.

Because we rated most of the interventions from very low-to-moderate evidence, possibilities of exploring other type of interventions could be multicomponent and multilevel interventions. Evidence-based multicomponent and multilevel interventions that address evidence-based MOV barriers may have a bigger overall effect than a single intervention. In fact, multiple studies have shown that multicomponent and multilevel interventions may significantly increase vaccine uptake [1,57,58]. Even though only one study assessed the effects of such interventions, our findings have proven the effectiveness of multicomponent interventions in reducing MOVs in children under age five.

Even though this is the first comprehensive systematic review and meta-analysis assessing interventions reducing MOVs in children under age five, this study has multiple limitations. First, most of the studies were conducted in high-income countries, making it challenging to integrate the results in resource-limited countries, more particularly in interventions such as education, integration of a pharmacist in children under age five immunisation programmes, and combined vaccines. Given the wide range of time periods covered by this review and the significant variation in health system contexts between high-income and resource-limited countries, our pooled estimates should be considered with caution to any specific real-world environment without careful consideration of local immunisation policies, and potential MOV barriers. Even through the comparison of the changing landscape of EIRs amongst the limited number of included studies, emerging technologies such as AI can be used to constantly enhance EIR interventions, which can change and improve over time with respect to functionality and public health impact. Furthermore, the small number of studies included in most of the meta-analyses reduced the statistical power, made the work prone to publication bias, intervention heterogeneity, and differences in outcome measurement, and limited generalisability. The high heterogeneity across studies might be attributed to participant characteristics, different intervention durations, and different types of vaccines as shown in the Supplemental Material S2. Moreover, the high heterogeneity may be due to both the design of interventions and the sociocultural contexts in which they were implemented. Importantly, included studies were from a wide variety of settings, including high-income countries such as the United States to middle- and low-income countries such as Nigeria, South Sudan, Sudan, South Africa, Pakistan and India. These settings differ not simply in economic and structural terms, but also culturally, socially and politically. These discrepancies will certainly have implications for parental beliefs about vaccination, faith in healthcare systems, acceptability of public health communications and response to specific intervention approaches. Evidence suggests that culturally tailored vaccination uptake interventions are generally more successful than standardised approaches [59]. Thus, the heterogeneity reported across the included MOV studies is likely to reflect not only changes in intervention design, but also disparities in underlying cultural and health system contexts. This represents another limitation when interpreting the overall effectiveness of MOV intervention in children under age five.

## 5. Conclusions

In summary, health personnel and caregivers’ education, EIRs and alerts, integrated delivery of health interventions, pharmacist integration in immunisation programmes, multicomponent interventions, combined vaccines, and financial incentives were effective in reducing MOVs in children under age five. However, this conclusion should be taken in a context of the very low to moderate grade of evidence and limited studies. Furthermore, most of the studies were conducted in high-income countries, limiting the integration of the review results in resource-limited countries where MOV prevalence remains high. In view of this study’s results, well-designed interventional studies related to MOVs in children under age five are needed in resource-limited countries. Furthermore, exploring multicomponent and multilevel interventions may be a useful tool to study more effective MOV interventions. Multicomponent and multilevel interventions often produce a greater overall effect than a single intervention. However, educational interventions may be the backbone of such combinations as they may target multiple MOV barriers.

## Data Availability

The original contributions presented in this study are included in the article/Supplementary Materials. Further inquiries can be directed to the corresponding author.

## Supplementary Materials

The following supporting information can be downloaded at: https://www.mdpi.com/article/10.3390/vaccines14060505/s1. Supplemental Material S1: Electronic search strategy; Supplemental Material S2: Table showing the reasons of exclusion [46,60,61,62,63,64,65,66,67,68,69,70,71,72,73,74,75,76,77]; Supplemental Material S3: Sensitivity analysis and other analysis; Supplemental Material S4: PRISMA 2020 checklist [16]. Figure S1: Leave-one-out meta-analysis showing education interventions. Figure S2: Effectiveness of EIRs and alerts interventions in reducing MOVs in under-five children: high-income vs. resource-limited countries. Figure S3: Effectiveness of EIRs and alerts interventions in reducing MOV in under-five children: High income vs. Resource-limited countries. Figure S4: Effectiveness of EIRs and alerts interventions in reducing MOV in under-five children: EIRs below and above year 2010. Figure S5: Leave-one-out meta-analysis showing of SMS and phone call reminder interventions. Figure S6: Leave-one-out meta-analysis showing integrated delivery of health interventions.

## Abbreviations

AI: artificial intelligence, CINAHL: Cumulative Index to Nursing and Allied Health Literature, EIRs: electronic immunisation records, IMCI: Integrated Management of Childhood Illness, MOVs: missed opportunities for vaccination, NRSIs: non-randomised studies of interventions, PRISMA: Preferred Reporting Items for Systematic reviews and Meta-Analyses, RCTs: randomised controlled trials, RRs: risk ratios, SAGE: Strategic Advisory Group of Experts, SMS: Short Message Service, VPDs: Vaccine Preventable Diseases; WHO: World Health Organization.

## Appendix A

**Table A1:** Table of characteristics of included studies.

Study ID	Country	Study Design	Population	Interventions	Duration	Tc	Nc	Ti	Ni	Vaccine Type
Davis et al., 2003 [28]	USA	Quasi-experimental study	12–24 months	Monovalent vs. combined vaccines	12 months	3424	1883	2774	1103	HepB and Hib
Oladeji et al., 2019 [26]	South Sudan	Community- and health facility-based interventional study	Under 5 years	IMCI	12 months	3203	1442	5791	1564	BCG, OPV, PENTA, and MCV
Szilagyi et al., 1996 [35]	USA	Randomised control trial	0 to 2 years	IMCI	12 months	994	895	994	597	Not specified
Harrison et al., 1993 [34]	South Africa	Quasi-experimental study	2 to 5 years	IMCI	Not specified	178	63	285	32	Not specified
Kolawole et al., 2021 [37]	Nigeria	Quasi-experimental study	Infants less than 2 years	SMS reminder	Not specified	136	81	132	7	PENTA1, PENTA 2, and PENTA 3)
Eze et al., 2023 [36]	Nigeria	Quasi-experimental study	Less than 8 months	Mobile phone reminders and recalls	4 weeks	145	32	145	13	BCG, OPV0, HB0, OPV1, PENTA1, PCV1
Fiks et al., 2007 [18]	USA	Quasi-experimental study	Less than 24 months	Electronic record and reminders	24 months	1548	816	1669	323	BCG, DTaP, Penta, Hib, PCV, OPV IPV, HepB, VAR, and MCV
Deivanayagam et al., 1994 [29]	India	Quasi-experimental study	Under 2 years	Education and awareness of immunisation among health personnel	12 months	1165	177	1165	91	BCG, DPT, OPV, and MCV
Minkovitz et al., 2001 [27]	USA	Randomised control trial	3 years or younger	Educated the acute care team monthly	12 months	213	65	295	76	DTP, Polio, MCV, HepB, and HiB.
Skull et al., 1999 [46]	USA	Quasi-experimental study	7 years	Education of staff and the introduction of prompts to record vaccination status	4 weeks	423	84	446	6	Not specified
Wood et al., 1998 [31]	USA	Randomised control trial	Under 2 years	Parent education and case management (CM)	2 weeks	126	68	129	61	DTP, Polio, MCV, and HiB
Sabnis et al., 2003 [32]	USA	Nonrandomised, before and after study	Children 36 months or younger	Effect of education, feedback, and provider prompts	Not specified	352	173	344	45	DTP, Polio, MCV, and HiB.
Hughart et al., 1998 [30]	USA	Pre- and post-intervention	Children aged 42 days to 9 months	Consultation–education sites	12 months	446	152	499	188	DTP, Polio, MCV, and HiB.
Stille et al., 2001 [33]	USA	Randomised control trial	Children aged 7 months	Educational intervention initiated at the first well childcare visit	7 months	159	58	156	28	DTP, Polio, Hib, and HepB.
Haas-Gehres et al., 2014 [21]	USA	A retrospective, quasi-experimental review	Less than 5 years	Full-time ambulatory clinical pharmacist integrated into the health care team	3 months	3044	892	3057	312	DTaP, IPV, Hib, HepB, RV, MCV, VAR, PCV13, hepa A, MenACW, tetanus, diphtheria, Tdap, and Td.
Wise et al., 2017 [20]	USA	A retrospective, quasi-experimental review	Less than 5 years	Full-time clinical pharmacist	3 months	504	51	504	30	DTaP, IPV, Hib, HepB, RV, MCV, VAR, PCV13, hepa A, MenACW, tetanus, diphtheria, Tdap, and Td.
Irigoyen et al., 2006 [45]	USA	Randomised control trial	Children aged 6 weeks–15 months	Every week, two reminder groups needed a repeat reminder for the previous dose or a reminder for a new dose. The reminders consisted of registry-generated postcards with the photograph of a baby and a standard bilingual English/Spanish message	6 months	931	140	920	138	DTP, Polio, MCV, and HiB.
Shaw et al., 2000 [44]	USA	Prospective randomised, controlled trial.	Under-five children	The intervention group received education based on manual prompts of immunisations due	2 weeks	328	71	298	34	All four doses of DPT
Siddiqi et al., 2023 [41]	Pakistan and Bangladesh	Mixed methods study design	Children aged less than 2 years	Mobile-based immunisation decision support system (iDSS) to automatically construct age-appropriate vaccination schedules for children. iDSS includes other digital applications or platforms, web-based or mobile-based electronic immunisation registries (EIRs)	Nine months	13,039	494	4922	89	BCG Penta, PCV, OPV IPV, and MCV
Koffi et al., 2011 [40]	Côte d’Ivoire	Quasi-experimental study	Under-five children	Integrating vaccination in primary health care program	Not specified	100	54	114	5	Not specified
Stephens et al., 2021 [43]	US	Randomised cluster–cross-over pragmatic clinical trial	Less than 12 months	EHR-based immunisation alert within the Allscripts Sunrise Clinical Manager (SCM) Ambulatory application. Using the configuration tools in the EHR, each site had four phases each lasting 3 months, two for which the reminder was “on” and two for which it was “off”	Three months	1096	324	1096	273	DTaP, IPV, Hib, HepB, RV, MCV, VAR, PCV13, hepa A, MenACW, tetanus, diphtheria, Tdap, Td
Stephens et al., 2021 [43]	US	Randomised cluster–cross-over pragmatic clinical trial	12–23 months	EHR-based immunisation alert within the Allscripts Sunrise Clinical Manager (SCM) Ambulatory application. Using the configuration tools in the EHR, each site had four phases each lasting 3 months, two for which the reminder was “on” and two for which it was “off”	Three months	1096	737	1096	669	DTaP, IPV, Hib, HepB, RV, MCV, VAR, PCV13, hepa A, MenACWY, tetanus, diphtheria, Tdap, Td
Stephens et al., 2021 [43]	US	Randomised cluster–cross-over pragmatic clinical trial	2–3 years	EHR-based immunisation alert within the Allscripts Sunrise Clinical Manager (SCM) Ambulatory application. Using the configuration tools in the EHR, each site had four phases each lasting 3 months, two for which the reminder was “on” and two for which it was “off”	Three months	1096	373	1096	334	DTaP, IPV, Hib, HepB, RV, MCV, VAR, PCV13, hepa A, MenACWY, tetanus, diphtheria, Tdap, Td
Rodewald et al., 1999 [42]	US	Randomised, controlled trial	0 to 12 months	Prompting-only/education	Two 18-month	767	139	801	145	DTP, Polio, MMR, and HiB.
Rodewald et al., 1999 [42]	US	Randomised, controlled trial	0 to 12 months	Tracking/outreach-only/the use of postcards and telephone calls	Two 18-month	767	139	715	98	DTP, Polio, MMR, and HiB.
Rodewald et al., 1999 [42]	US	Randomised, controlled trial	0 to 12 months	Combined tracking/outreach with prompting	Two 18-month	767	139	732	82	DTP, Polio, MMR, and HiB.
Loevinsohn et al., 1992 [48]	Sudan	Randomised cross-over study	Less than one-year children	Intervention A: more accessible site of vaccination Intervention B: referral for vaccination	Two weeks	129	19	141	17	Vaccines in immunisation schedule in less than one year
Fairbrother et al., 1997 [38]	US	Pre-intervention and post-intervention study design	Children aged 3 to 35 months	Free vaccination programme	12 months	173	35	528	106	DTP, Polio, and MMR.
Minkovitz et al., 1999 [39]	US	Randomised controlled trial.	Children aged 3 to 24 months	Families in the experimental group were penalised financially for failing to verify that their children received vaccinations	Two years	864	259	911	238	DTP, polio, and MMR
Secor et al., 2022 [47]	Tanzania	Population-based cohort study	Under-five children	EIRs can identify missed opportunities for vaccination depending: short vs. long EIR duration use	0–5 months vs. 6–11 months	557,674	130,611	557,674	50,638	BCG, Penta, polio, PCV, rotavirus, and MCV
Secor et al., 2022 [47]	Tanzania	Population-based cohort study	Under-five children	EIRs can identify missed opportunities for vaccination depending: short vs. long EIR duration use	1 year vs. ≥2 years	557,674	37,625	557,674	6576	BCG, Penta, polio, PCV, rotavirus, and MCV

## References

[1] Siddiqui F.A., Padhani Z.A., Salam R.A., Aliani R., Lassi Z.S., Das J.K., Bhutta Z.A. (2022). Interventions to Improve Immunization Coverage Among Children and Adolescents: A Meta-Analysis. Pediatrics.

[2] The Lancet Infectious Diseases (2017). The Imperative of Vaccination. Lancet Infect. Dis..

[3] Jacobson Vann J.C., Jacobson R.M., Coyne-Beasley T., Asafu-Adjei J.K., Szilagyi P.G. (2018). Patient Reminder and Recall Interventions to Improve Immunization Rates. Cochrane Database Syst. Rev..

[4] WHO (2016). Meeting of the Strategic Advisory Group of Experts on immunization, April 2016–conclusions and recommendations. Wkly. Epidemiol. Rec..

[5] Jaca A., Mathebula L., Iweze A., Pienaar E., Wiysonge C.S. (2018). A Systematic Review of Strategies for Reducing Missed Opportunities for Vaccination. Vaccine.

[6] Sridhar S., Maleq N., Guillermet E., Colombini A., Gessner B.D. (2014). A Systematic Literature Review of Missed Opportunities for Immunization in Low-and Middle-Income Countries. Vaccine.

[7] Hutchins S.S., Jansen H., Robertson S.E., Evans P., Kim-Farley R.J. (1993). Studies of Missed Opportunities for Immunization in Developing and Industrialized Countries. Bull. World Health Organ..

[8] Tamuzi J.L., Katoto P.D.M.C., Ndwandwe D.E., Wiysonge C.S., Nyasulu P.S. (2025). Prevalence of Missed Opportunities for Vaccination (MOV) Indicators among Children Aged 12–23 Months in Sub-Saharan African Countries: An Individual-Level Meta-Analysis of DHS and MICS National Household Data Surveys. Hum. Vaccines Immunother..

[9] Jejaw M., Tafere T.Z., Tiruneh M.G., Hagos A., Teshale G., Tilahun M.M., Negash W.D., Demissie K.A. (2025). Three in Four Children Age 12–23 Months Missed Opportunities for Vaccination in Sub-Saharan African Countries: A Multilevel Mixed Effect Analysis of Demographic Health and Surveys 2016–2023. BMC Public Health.

[10] Borras-Bermejo B., Panunzi I., Bachy C., Gil-Cuesta J. (2022). Missed Opportunities for Vaccination (MOV) in Children up to 5 Years Old in 19 Médecins Sans Frontières-Supported Health Facilities: A Cross-Sectional Survey in Six Low-Resource Countries. BMJ Open.

[11] Oyo-Ita A., Oduwole O., Arikpo D., Effa E.E., Esu E.B., Balakrishna Y., Chibuzor M.T., Oringanje C.M., Nwachukwu C.E., Wiysonge C.S. (2023). Interventions for Improving Coverage of Childhood Immunisation in Low- and Middle-Income Countries. Cochrane Database Syst. Rev..

[12] World Health Organization (2017). Methodology for the Assessment of Missed Opportunities for Vaccination.

[13] Tampi M., Carrasco-Labra A., O’Brien K.K., Velandia-González M., Brignardello-Petersen R. (2022). Systematic Review on Reducing Missed Opportunities for Vaccinations in Latin America. Rev. Panam. Salud Publica.

[14] World Health Organization (2017). Planning Guide to Reduce Missed Opportunities for Vaccination.

[15] Dadanova G., Horth R., Kubatova A., Ishenapysova G., Otorbaeva D., Nabirova D. (2025). Missed Opportunities for Vaccination among Healthcare-Seeking Children: A Cross-Sectional Study in Bishkek, Kyrgyzstan, 2023. BMJ Public Health.

[16] Page M.J., McKenzie J.E., Bossuyt P.M., Boutron I., Hoffmann T.C., Mulrow C.D., Shamseer L., Tetzlaff J.M., Akl E.A., Brennan S.E. (2021). The PRISMA 2020 Statement: An Updated Guideline for Reporting Systematic Reviews. BMJ.

[17] Johnson K.B., Davison C.L. (2004). Information Technology: Its Importance to Child Safety. Ambul. Pediatr..

[18] Fiks A.G., Grundmeier R.W., Biggs L.M., Localio A.R., Alessandrini E.A. (2007). Impact of Clinical Alerts within an Electronic Health Record on Routine Childhood Immunization in an Urban Pediatric Population. Pediatrics.

[19] World Health Organization (2017). Guideline: Assessing and Managing Children at Primary Health-Care Facilities to Prevent Overweight and Obesity in the Context of the Double Burden of Malnutrition: Updates for the Integrated Management of Childhood Illness (IMCI).

[20] Wise K.A., Sebastian S.J., Haas-Gehres A.C., Moore-Clingenpeel M.D., Lamberjack K.E. (2017). Pharmacist Impact on Pediatric Vaccination Errors and Missed Opportunities in the Setting of Clinical Decision Support. J. Am. Pharm. Assoc..

[21] Haas-Gehres A., Sebastian S., Lamberjack K. (2014). Impact of Pharmacist Integration in a Pediatric Primary Care Clinic on Vaccination Errors: A Retrospective Review. J. Am. Pharm. Assoc..

[22] Higgins J.P.T., Thomas J., Chandler J., Cumpston M., Li T., Page M.J., Welch V.A. (2024). Cochrane Handbook for Systematic Reviews of Interventions.

[23] Guyatt G.H., Oxman A.D., Vist G.E., Kunz R., Falck-Ytter Y., Alonso-Coello P., Schünemann H.J. (2008). GRADE: An Emerging Consensus on Rating Quality of Evidence and Strength of Recommendations. BMJ.

[24] Balshem H., Helfand M., Schünemann H.J., Oxman A.D., Kunz R., Brozek J., Vist G.E., Falck-Ytter Y., Meerpohl J., Norris S. (2011). GRADE Guidelines: 3. Rating the Quality of Evidence. J. Clin. Epidemiol..

[25] Brożek J., Guyatt G., Oxman A. (2013). GRADE Handbook.

[26] Oladeji O., Campbell P., Jaiswal C., Chamla D., Oladeji B., Ajumara C.O., Minguiel L.M., Senesie J. (2019). Integrating Immunisation Services into Nutrition Sites to Improve Immunisation Status of Internally Displaced Persons’ Children Living in Bentiu Protection of Civilian Site, South Sudan. Pan Afr. Med. J..

[27] Minkovitz C.S., Belote A.D., Higman S.M., Serwint J.R., Weiner J.P. (2001). Effectiveness of a Practice-Based Intervention to Increase Vaccination Rates and Reduce Missed Opportunities. Arch. Pediatr. Adolesc. Med..

[28] Davis R.L., Coplan P., Mell L., Black S., Shinefield H., Lewis E. (2003). Impact of the Introduction of a Combined Haemophilus B Conjugate Vaccine and Hepatitis B Recombinant Vaccine on Vaccine Coverage Rats in a Large West Coast Health Maintenance Organization. Pediatr. Infect. Dis. J..

[29] Deivanayagam N., Nedunchelian K., Mala N., Ashok T.P., Rathnam S.R., Ahmed S.S. (1995). Missed Opportunities for Immunization in Children under 2 Years Attending an Urban Teaching Hospital. Indian Pediatr..

[30] Hughart N., Holt E., Rosenthal J., Ross A., Jones A., Keane V., Vivier P., Guyer B. (1998). Effectiveness of Pediatric Practice Consultation on Missed Opportunities for Immunization. J. Urban Health.

[31] Wood D., Schuster M., Donald-Sherbourne C., Duan N., Mazel R., Halfon N. (1998). Reducing Missed Opportunities to Vaccinate during Child Health Visits. How Effective Are Parent Education and Case Management?. Arch. Pediatr. Adolesc. Med..

[32] Sabnis S.S., Pomeranz A.J., Amateau M.M. (2003). The Effect of Education, Feedback, and Provider Prompts on the Rate of Missed Vaccine Opportunities in a Community Health Center. Clin. Pediatr..

[33] Stille C.J., Christison-Lagay J., Bernstein B.A., Dworkin P.H. (2001). A Simple Provider-Based Educational Intervention to Boost Infant Immunization Rates: A Controlled Trial. Clin. Pediatr..

[34] Harrison D., Barron P., Glass B., Sonday S., vd Heyde Y. (1993). Far Fewer Missed Opportunities for Inununisation in an Integrated Child Health Service. S. Afr. Med. J..

[35] Szilagyi P.G., Rodewald L.E., Humiston S.G., Pollard L., Klossner K., Jones A.M., Barth R., Woodin K.A. (1996). Reducing Missed Opportunities for Immunizations. Easier Said than Done. Arch. Pediatr. Adolesc. Med..

[36] Eze U., Tanimowo S., Ajadi A., Okeke C., Ohazurike C., Uzochukwu B.C. (2023). Assessment of the Burden and Factors Associated with Missed Opportunities for Vaccination in a South-Western State of Nigeria: Toward Immunization Agenda 2030. Niger. J. Clin. Pract..

[37] Kolawole T.O., Sotunsa J.O. (2021). Effect of Short Message Service on Prevention of Missed Childhood Immunization Among Mothers Attending Immunization Clinics in Selected Hospitals in Lagos State, Nigeria. Int. J. Med. Nurs. Health Sci..

[38] Fairbrother G., Friedman S., Hanson K.L., Butts G.C. (1997). Effect of the Vaccines for Children Program on Inner-City Neighborhood Physicians. Arch. Pediatr. Adolesc. Med..

[39] Minkovitz C., Holt E., Hughart N., Hou W., Thomas L., Dini E., Guyer B. (1999). The Effect of Parental Monetary Sanctions on the Vaccination Status of Young Children: An Evaluation of Welfare Reform in Maryland. Arch. Pediatr. Adolesc. Med..

[40] Koffi K., Menin M., Andoh A.T. (2011). The Efficacy of Integrating Vaccination in Primary Heath Care Program to Prevent Missed Opportunities of Vaccination in Ivory Cost. Rech. Soins Infirm..

[41] Siddiqi D.A., Ali R.F., Shah M.T., Dharma V.K., Khan A.A., Roy T., Chandir S. (2023). Evaluation of a Mobile-Based Immunization Decision Support System for Scheduling Age-Appropriate Vaccine Schedules for Children Younger than 2 Years in Pakistan and Bangladesh: Lessons from a Multisite, Mixed Methods Study. JMIR Pediatr. Parent..

[42] Rodewald L.E., Szilagyi P.G., Humiston S.G., Barth R., Kraus R., Raubertas R.F. (1999). A Randomized Study of Tracking with Outreach and Provider Prompting to Improve Immunization Coverage and Primary Care. Pediatrics.

[43] Stephens A.B., Wynn C.S., Hofstetter A.M., Kolff C., Pena O., Kahn E., Dasgupta B., Natarajan K., Vawdrey D.K., Lane M.M. (2021). Effect of Electronic Health Record Reminders for Routine Immunizations and Immunizations Needed for Chronic Medical Conditions. Appl. Clin. Inform..

[44] Shaw J.S., Samuels R.C., Larusso E.M., Bernstein H.H. (2000). Impact of an Encounter-Based Prompting System on Resident Vaccine Administration Performance and Immunization Knowledge. Pediatrics.

[45] Irigoyen M.M., Findley S., Wang D., Chen S., Chimkin F., Pena O., Mendonca E. (2006). Challenges and Successes of Immunization Registry Reminders at Inner-City Practices. Ambul. Pediatr..

[46] Skull S., Krause V., Roberts L., Dalton C. (1999). Evaluating the Potential for Opportunistic Vaccination in a Northern Territory Hospital. J. Paediatr. Child Health.

[47] Secor A.M., Mtenga H., Richard J., Bulula N., Ferriss E., Rathod M., Ryman T.K., Werner L., Carnahan E. (2022). Added Value of Electronic Immunization Registries in Low-and Middle-Income Countries: Observational Case Study in Tanzania. JMIR Public Health Surveill..

[48] Loevinsohn B.P., Gareaballah E. (1992). Missed Opportunities for Immunization during Visits for Curative Care: A Randomized Cross-over Trial in Sudan. Bull. World Health Organ..

[49] Hu Y., Chen Y., Wang Y., Liang H. (2018). Evaluation of Potentially Achievable Vaccination Coverage of the Second Dose of Measles Containing Vaccine with Simultaneous Administration and Risk Factors for Missed Opportunities among Children in Zhejiang Province, East China. Hum. Vaccines Immunother..

[50] Li P.-P., Dai Y.-T., Zhang B.-L., Ye L.-X. (2021). The Impact of Simultaneous Vaccine Administration on Missed Opportunities for the Second Dose of Seasonal Influenza Vaccine in Children. Chin. Prev. Med..

[51] Tamuzi J.L., Katoto P.D.M.C., Sakala D.Y., Wiysonge C.S., Nyasulu P.S. (2026). Determinants of Missed Opportunities for Vaccination (MOVs) Indicators Among Children Aged 12–23 Months in Sub-Saharan African Countries: A Multilevel Analysis of Survey Data. Vaccines.

[52] Gilano G., Sako S., Molla B., Dekker A., Fijten R. (2024). The Effect of mHealth on Childhood Vaccination in Africa: A Systematic Review and Meta-Analysis. PLoS ONE.

[53] Taseen S., Yousafzai M.T., Qureshi M.F.H. (2025). Harnessing Artificial Intelligence and Digital Technology for Enhancing Routine Immunization among Zero-Dose Children. Digit. Health.

[54] Acharya K., Lacoul M., Bietsch K. (2019). Factors Affecting Vaccination Coverage and Retention of Vaccination Cards in Nepal.

[55] Uganda National Institute of Public Health (2021). Reduce Missed Measles Vaccination Opportunities to Save Our Children: Policy Brief.

[56] WHO (2026). Reducing Missed Opportunities for Vaccination (MOV).

[57] Loskutova N.Y., Smail C., Callen E., Staton E.W., Nazir N., Webster B., Pace W.D. (2020). Effects of Multicomponent Primary Care-Based Intervention on Immunization Rates and Missed Opportunities to Vaccinate Adults. BMC Fam. Pract..

[58] Parsekar S.S., Vadrevu L., Jain M., Menon S., Taneja G. (2024). Interventions Addressing Routine Childhood Immunization and Its Behavioral and Social Drivers. Front. Public Health.

[59] Ekezie W., Igein B., Varughese J., Butt A., Ukoha-Kalu B.O., Ikhile I., Bosah G. (2024). Vaccination Communication Strategies and Uptake in Africa: A Systematic Review. Vaccines.

[60] Jong K., Sikora C., MacDonald S. (2021). Childhood immunization appointment reminders and recalls: Strengths, weaknesses and opportunities to increase vaccine coverage. Public Health.

[61] Fiks A.G., Hunter K.F., Localio A.R., Grundmeier R.W., Bryant-Stephens T., Luberti A.A., Bell L.M., Alessandrini E.A. (2009). Impact of Electronic Health Record-Based Alerts on Influenza Vaccination for Children with Asthma. Pediatrics.

[62] Sawyer M.H., A Pung M., Ho S., Chiang J.C., De Guire M., Fontanesi J., Nader P.R. (1999). Change in Immunization Missed Opportunities by Providers Following an Intensive Intervention. Pediatr. Res..

[63] Daley M.F., Barrow J., Pearson K., Crane L.A., Gao D., Stevenson J.M., Berman S., Kempe A. (2004). Identification and Recall of Children with Chronic Medical Conditions for Influenza Vaccination. Pediatrics.

[64] Daisy E. The Role of Digital Registries in Reducing Missed Opportunities for Vaccination in Nigerian Infants. https://www.researchgate.net/publication/392736272_The_Role_of_Digital_Registries_in_Reducing_Missed_Opportunities_for_Vaccination_in_Nigerian_Infants.

[65] Dhaliwal B.K., Mathew J.L., Obiagwu P.N., Hill R., Wonodi C.B., Best T., Shet A. (2024). Addressing Missed Opportunities for Vaccination among Children in Hospitals: Leveraging the P-Process for Health Communication Strategies. Vaccines.

[66] A Adamu A., A Uthman O., A Gadanya M., Wiysonge C.S. (2019). Implementation and evaluation of a collaborative quality improvement program to improve immunization rate and reduce missed opportunities for vaccination in primary health-care facilities: A time series study in Kano, Nigeria. Expert Rev. Vaccines.

[67] Tarca A.J., Lau G.T., Mascaro F., Clifford P., Campbell A.J., Taylor E. (2020). Pre- and post-intervention study examining immunisation rates, documentation, catch-up delivery and the impact of a dedicated immunisation service at a tertiary paediatric hospital. J. Paediatr. Child Health.

[68] Dombkowski K.J., Costello L., Dong S., Clark S.J. (2014). Using administrative claims to identify children with chronic conditions in a statewide immunization registry. Am. J. Manag. Care.

[69] Schlumberger M. (2023). Increasing the efficiency of a mobile EPI strategy using injectable polio vaccine in Africa. Med. Trop. Sante Int..

[70] Hicks P., Tarr G.A.M., Hicks X.P. (2007). Reminder Cards and Immunization Rates Among Latinos and the Rural Poor in Northeast Colorado. J. Am. Board Fam. Med..

[71] Fisker A.B., Martins J.S.D., Jensen A.M., Martins C., Aaby P., Thysen S.M. (2022). Health effects of utilising hospital contacts to provide measles vaccination to children 9–59 months—A randomised controlled trial in Guinea-Bissau. Trials.

[72] Vora S., Verber L., Potts S., Dozier T., Daum R.S. (2009). Effect of a novel birth intervention and reminder-recall on on-time immunization compliance in high-risk children. Hum. Vaccines.

[73] Appiah B., Gebretsadik L.A., Mamo A., Kmush B., Asefa Y., France C.R., Samman E., Alemayehu T., Abafogi M., Ahmed K. (2022). A 10+10+30 radio campaign is associated with increased infant vaccination and decreased morbidity in Jimma Zone, Ethiopia: A prospective, quasi-experimental trial. PLoS Glob. Public Health.

[74] Werk L.N., Diaz M.C., Cadilla A., Franciosi J.P., Hossain J. (2019). Promoting Adherence to Influenza Vaccination Recommendations in Pediatric Practice. J. Prim. Care Community Health.

[75] Schickedanz A., Perales L., Holguin M., Rhone-Collins M.M., Robinson H., Tehrani N., Smith L., Chung P.J., Szilagyi P.G. (2023). Clinic-Based Financial Coaching and Missed Pediatric Preventive Care: A Randomized Trial. Pediatrics.

[76] Ball T.M., Serwint J.R. (1996). Missed Opportunities for Vaccination and the Delivery of Preventive Care. Arch. Pediatr. Adolesc. Med..

[77] Elia S., Perrett K., Newall F. (2017). Providing opportunistic immunisations for at-risk inpatients in a tertiary paediatric hospital. J. Spéc. Pediatr. Nurs..

