# Supplementary material for: A Systematic Review and Meta-Analysis on the Effectiveness of Interventions in Reducing Missed Opportunities for Vaccination Among Children Under Age Five
Source: Vaccines (Basel). 2026 Jun 4;14(6):505. doi: 10.3390/vaccines14060505 (PMC13307740; doi:10.3390/vaccines14060505)
Supplement: Supplementary file 1 [file vaccines-14-00505-s001.zip › vaccines-4315608-supplementary.pdf]

Supplemental Materials: A systematic review and meta-analysis on the effectiveness of interventions in reducing missed opportunities for vaccination among children under five

Supplemental Material 1: Electronic search strategy

Number	Database	Electronic search strategy
1	Pubmed (Medline)	("Vaccination"[Mesh] OR "Immunization"[Mesh] OR vaccin*[tiab] OR immuni*[tiab]) AND ("Missed Opportunities"[tiab] OR "missed opportunity"[tiab] OR "missed opportunities for vaccination"[tiab]) AND ("Child"[Mesh] OR "Infant"[Mesh] OR child*[tiab] OR infant*[tiab] OR "under five"[tiab] OR "under 5"[tiab]) AND ("Health Education"[Mesh] OR "Reminder Systems"[Mesh] OR "Text Messaging"[Mesh] OR "Electronic Health Records"[Mesh] OR "Pharmacists"[Mesh] OR "Outreach Programs"[Mesh] OR "Combined Vaccines"[Mesh] OR "Financial Incentives"[Mesh] OR intervention*[tiab] OR education*[tiab] OR SMS[tiab] OR "text message*"[tiab] OR call*[tiab] OR reminder*[tiab] OR "electronic immunization register*"[tiab] OR pharmacist*[tiab] OR outreach[tiab] OR "combined vaccine*"[tiab] OR "integrated management of child immunization"[tiab] OR "integrated child immunization"[tiab] OR "financial incentive*"[tiab]) AND ("Randomized Controlled Trial"[Publication Type] OR "Clinical Trial"[Publication Type] OR randomized[tiab] OR randomised[tiab] OR "controlled trial"[tiab])
2	Scopus	(vaccin* OR immuni*) AND ("missed opportunity" OR "missed opportunities") AND (child* OR infant* OR "under five" OR "under 5") AND (intervention* OR education* OR SMS OR "text message*" OR call* OR reminder* OR "electronic immunization register*" OR "electronic immunisation register*" OR pharmacist* OR outreach OR "combined vaccine*" OR "integrated management of child immunization" OR "integrated child immunization" OR "financial incentive*") AND ("randomized controlled trial" OR "randomised controlled trial" OR "controlled trial" OR "clinical trial")
3	Web of Science	(vaccin* OR immuni*) AND ("missed opportunity" OR "missed opportunities") AND (child* OR infant* OR "under five" OR "under 5") AND (intervention* OR education* OR SMS OR "text

		message*" OR call* OR reminder* OR "electronic immunization register*" OR "electronic immunisation register*" OR pharmacist* OR outreach OR "combined vaccine*" OR "integrated management of child immunization" OR "integrated child immunization" OR "financial incentive*") AND ("randomized controlled trial" OR "randomised controlled trial" OR "controlled trial" OR "clinical trial")
4	CINAHL (EBSCOhost)	(MH "Vaccination+" OR MH "Immunization+" OR vaccin* OR vaccin* OR immuni*) AND ("missed opportunity" OR "missed opportunities") AND (MH "Child+" OR MH "Infant+" OR child* OR "under five") AND (MH "Health Education+" OR MH "Reminder Systems+" OR MH "Text Messaging+" OR MH "Electronic Health Records+" OR MH "Pharmacists+" OR MH "Outreach Programs+" OR MH "Combined Vaccines+" OR MH "Financial Incentives+" OR intervention* OR education* OR SMS OR reminder* OR "electronic immunization register*" OR "electronic immunization register*" OR pharmacist* OR outreach OR "combined vaccine*" OR "combined vaccine*" OR "integrated management of child immunization" OR AB "integrated management of child immunization" OR "financial incentive*" OR "financial incentive*") AND (MH "Randomized Controlled Trials" OR MH "Clinical Trials" OR randomi* OR "controlled trial" OR "controlled trial")
5	CENTRAL (Cochrane Central Register of Controlled Trials)	(vaccin* OR immuni*) AND ("missed opportunity" OR "missed opportunities") AND (child* OR infant* OR "under five" OR "under 5") AND (intervention* OR education* OR SMS OR "text message*" OR call* OR reminder* OR "electronic immunization register*" OR pharmacist* OR outreach OR "combined vaccine*" OR "integrated management of child immunization" OR "financial incentive*") AND (randomized OR randomised OR "controlled trial" OR "clinical trial")
6	Google Scholar	"missed opportunities for vaccination" AND (vaccination OR immunization) AND (children OR "under five") AND (intervention OR education OR SMS OR reminders OR outreach OR pharmacist OR "combined vaccines" OR "financial incentives") AND ("randomized controlled trial" OR "clinical trial")

Supplemental Material 2: Table showing the reasons of exclusion

N	Study ID	Reasons	Reference
1	Jong et al., 2021	Study design was an online survey.	[60]
2	Fiks et al., 2009	Children between 5 and 19 years of age were included	[61]
3	Skull et al., 1999	Children aged 7 years and above were included.	[46]
4	Sawyer et al., 1999	Full text was not available.	[62]
5	Daley et al., 2004	Impossible to draw the sample size of under-five children from children aged 6 to 72 months.	[63]
6	Daisy 2025	Full text was not available.	[64]
7	Dhaliwal et al., 2024	Ongoing research is still at the stage of study protocol.	[65]
8	Adamu et al., 2029	The study design was a time series analysis.	[66]
9	Taylor et al., 2021	The outcome of interest was not included.	[67]
10	Dombkowski et al., 2014	Full text not found	[68]
11	Schlumberger et al., 2023	The outcome of interest was not clearly quantified.	[69]
12	Hicks et al., 2007	The outcome of interest was not clearly quantified.	[70]
13	Fisker et al., 2022	The outcome of interest was not quantified.	[71]
14	Vora et al., 2009	The outcome of interest was not quantified.	[72]
15	Appiah et al., 2022	The outcome of interest was not quantified.	[73]
16	Werk et al., 2019	The outcome of interest was not clearly quantified.	[74]
17	Schickedan et al., 2023	The outcome of interest was not quantified.	[75]
18	Ball et al., 1996	The study design was a cross-sectional study	[76]
19	Elia et al., 2017	The outcome of interest was not quantified.	[77]

Supplemental Material 3: Sensitivity analysis and other analysis

Study	Leave-One-Out Meta-Analysis	RR	95%-CI	P-value	Tau2	Tau	I2
Omitting Deivanayagam et al., 1995		0.67	[0.46; 0.98]	0.0391	0.2391	0.4890	92.8%
Omitting Minkovitz et al., 2001		0.63	[0.43; 0.91]	0.0145	0.2380	0.4878	93.6%
Omitting Sabnis et al., 2003		0.75	[0.58; 0.96]	0.0235	0.0976	0.3124	85.6%
Omitting Hughart et al., 1998		0.60	[0.42; 0.85]	0.0036	0.1938	0.4403	91.1%
Omitting Shaw et al., 2000		0.67	[0.46; 0.97]	0.0352	0.2399	0.4898	93.4%
Omitting Stille et al., 2001		0.67	[0.46; 0.98]	0.0371	0.2342	0.4839	93.3%
Omitting Wood et al., 1998		0.62	[0.43; 0.90]	0.0127	0.2342	0.4839	93.5%
Omitting Rodewald et al., 1999a		0.61	[0.42; 0.87]	0.0071	0.2152	0.4639	93.0%
**Random effects model**		**0.65**	**[0.47; 0.91]**	**0.0114**	**0.2118**	**0.4603**	**92.6%**

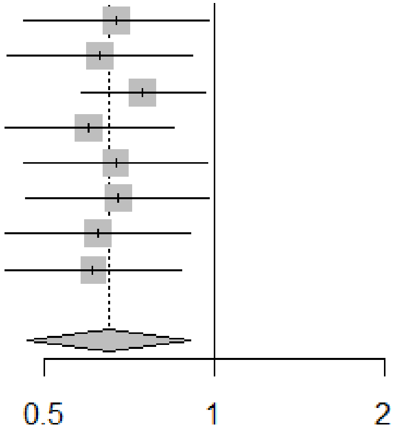

Supplemental Figure 1: Leave-one-out meta-analysis showing education interventions.

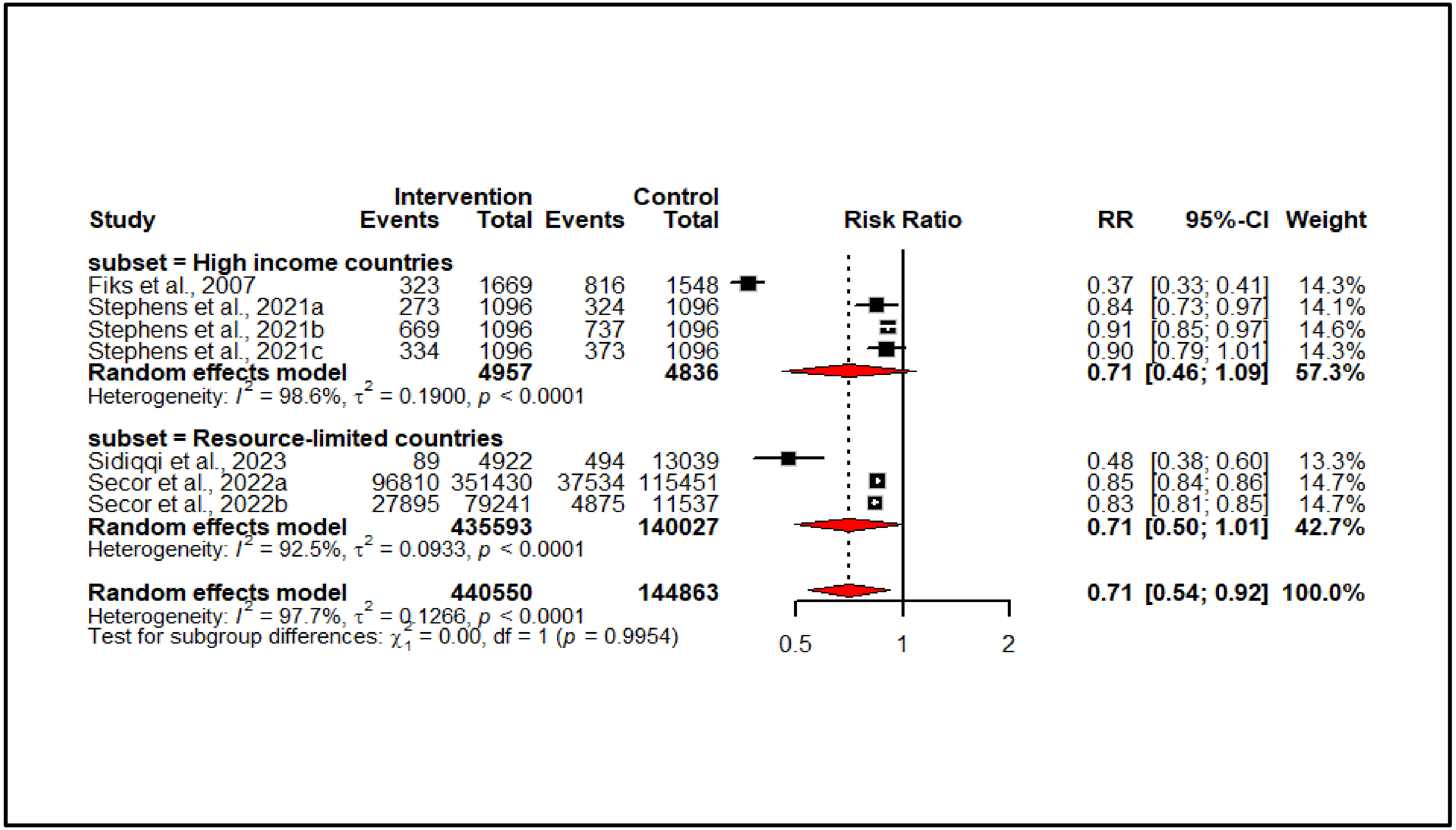

Supplemental Figure 2: Effectiveness of EIRs and alerts interventions in reducing MOVs in under-five children: high-income vs. resource-limited countries.

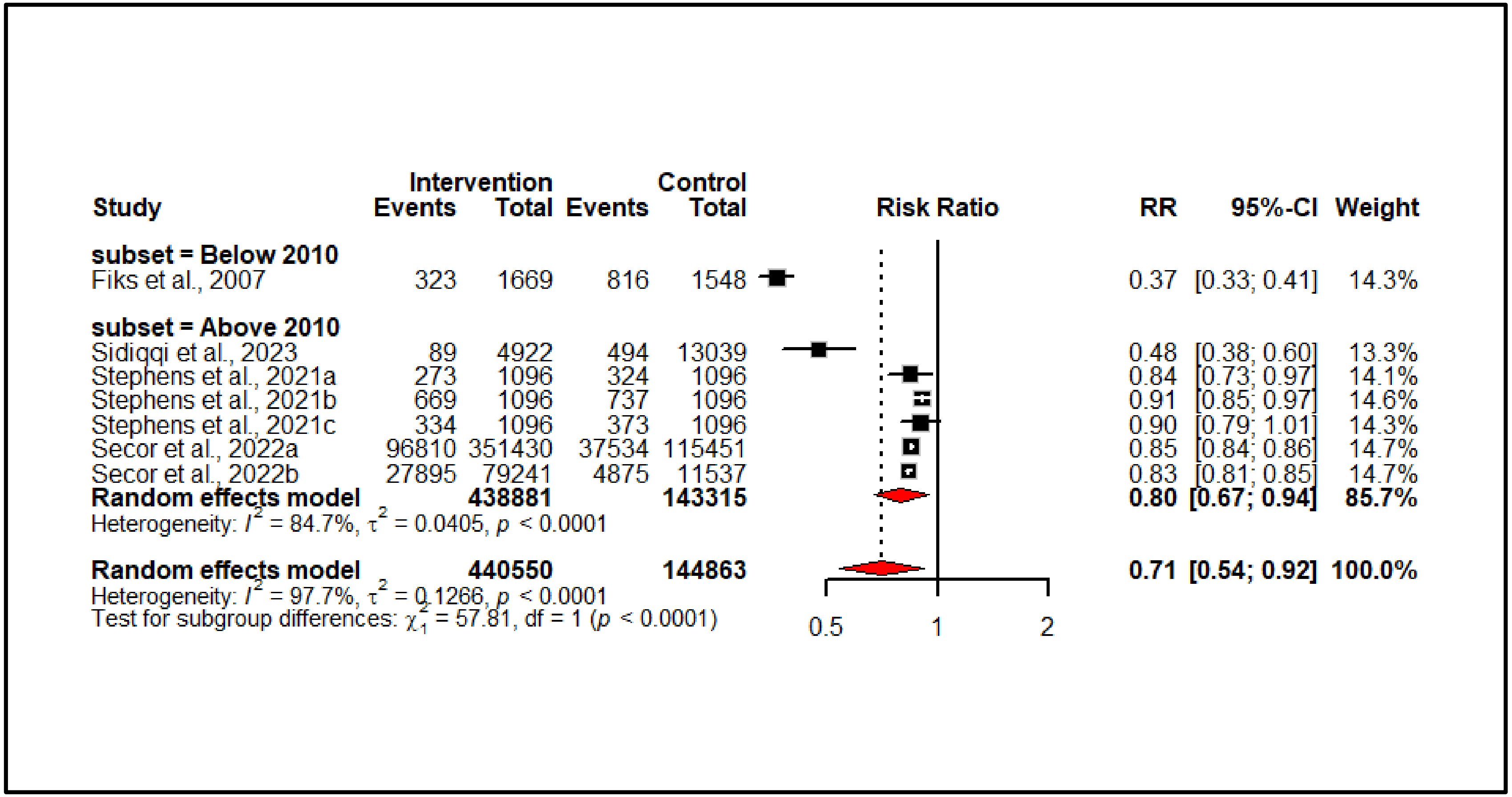

Supplemental Figure 3: Effectiveness of EIRs and alerts interventions in reducing MOV in under-five children: High income vs. Resource-limited countries

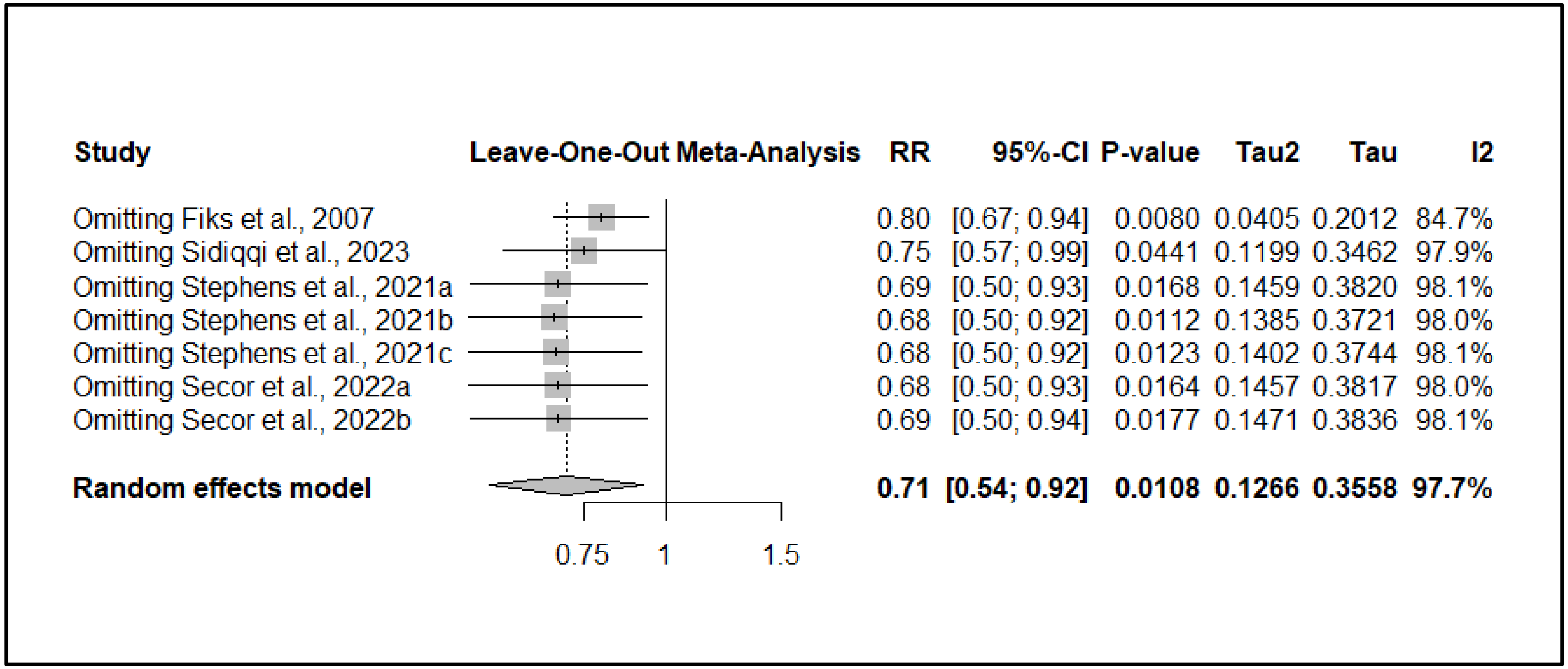

Supplemental Figure 4: Effectiveness of EIRs and alerts interventions in reducing MOV in under-five children: EIRs below and above year 2010.

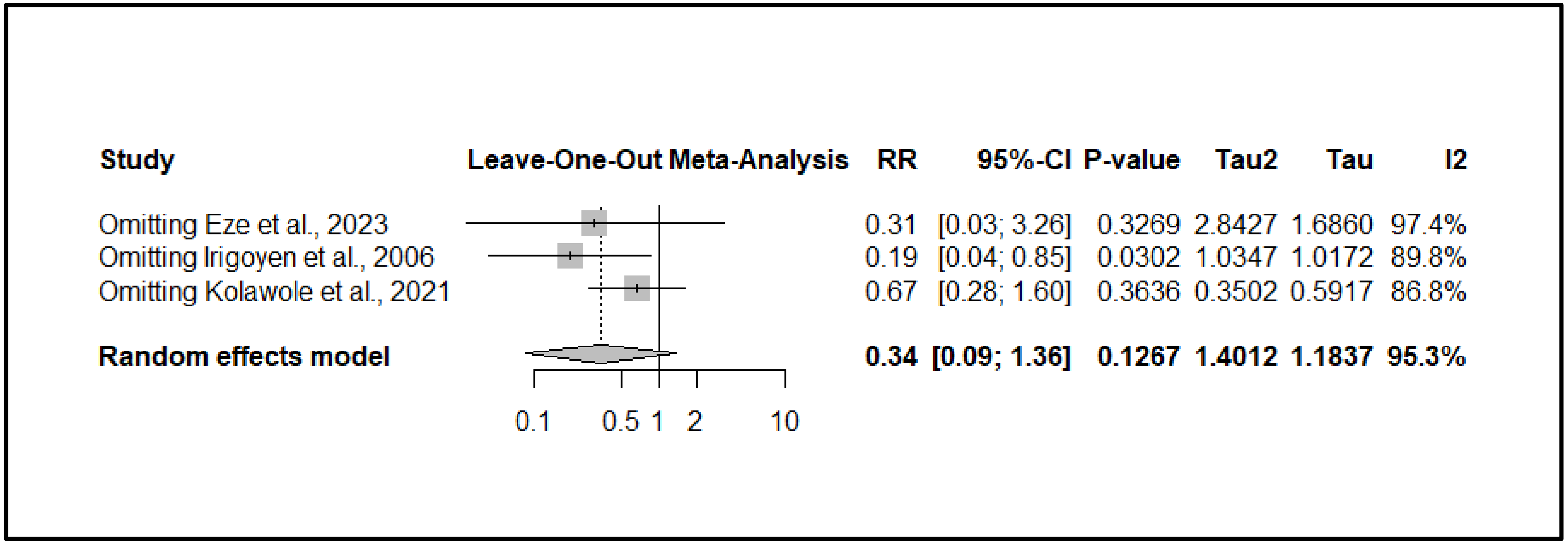

Supplemental Figure 5: Leave-one-out meta-analysis showing of SMS and phone call reminder interventions

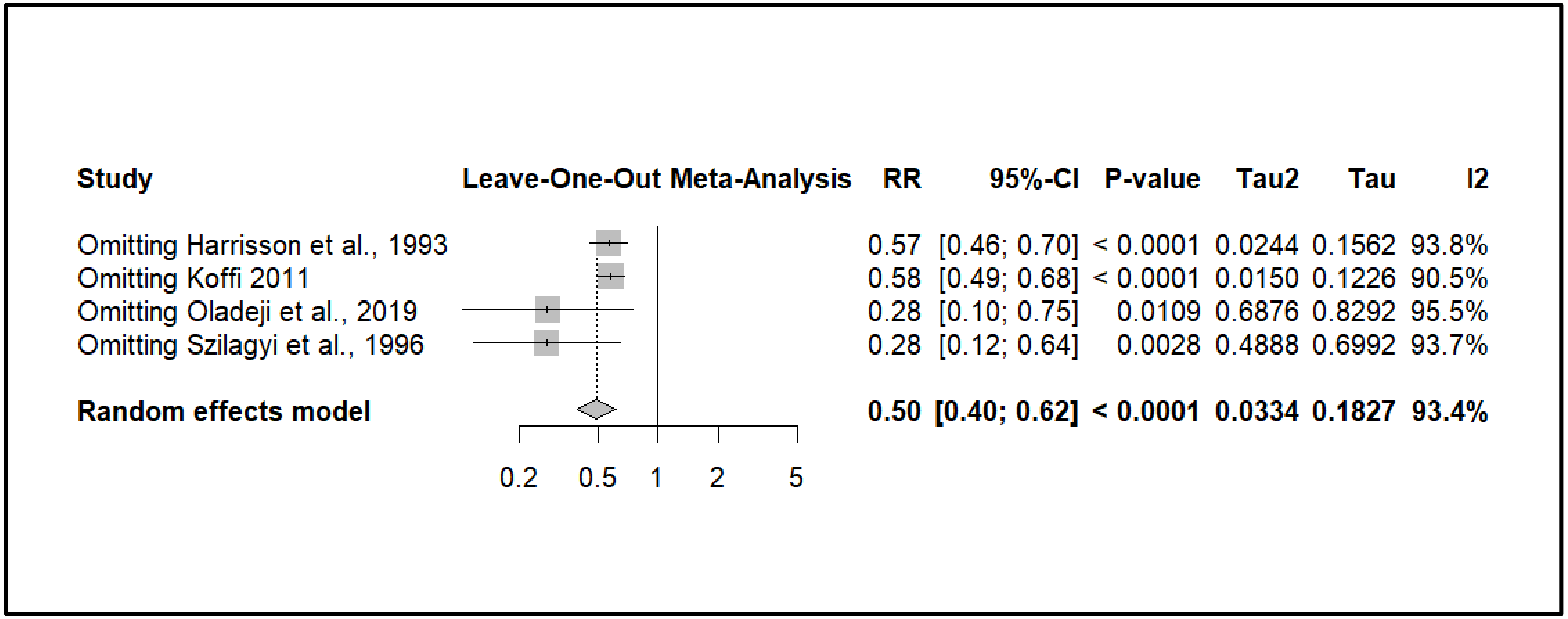

Supplemental Figure 6: Leave-one-out meta-analysis showing integrated delivery of health interventions.

Supplemental Material 4: PRISMA 2020 checklist [16]

Section and Topic	Item #	Checklist item	Location where item is reported
**TITLE**			
Title	1	Identify the report as a systematic review.	Page 1, line 2-4

Section and Topic	Item #	Checklist item	Location where item is reported
**ABSTRACT**			
Abstract	2	See the PRISMA 2020 for Abstracts checklist.	Page 1-2, lines 23 - 47
**INTRODUCTION**			
Rationale	3	Describe the rationale for the review in the context of existing knowledge.	Page 2-3, lines 50 - 92
Objectives	4	Provide an explicit statement of the objective(s) or question(s) the review addresses.	Page 2, lines 91-92; Page 3, lines 98-103
**METHODS**			
Eligibility criteria	5	Specify the inclusion and exclusion criteria for the review and how studies were grouped for the syntheses.	Page 3, lines 114 - 124
Information sources	6	Specify all databases, registers, websites, organisations, reference lists and other sources searched or consulted to identify studies. Specify the date when each source was last searched or consulted.	Page 3, lines 105-113
Search strategy	7	Present the full search strategies for all databases, registers and websites, including any filters and limits used.	Supplemental materials
Selection process	8	Specify the methods used to decide whether a study met the inclusion criteria of the review, including how many reviewers screened each record and each report retrieved, whether they worked independently, and if applicable, details of automation tools used in the process.	Page 3, lines 114 – 124
Data collection process	9	Specify the methods used to collect data from reports, including how many reviewers collected data from each report, whether they worked independently, any processes for obtaining or confirming data from study investigators, and if applicable, details of automation tools used in the process.	Page 3, lines 12-113; Page 4, lines 166-171
Data items	10a	List and define all outcomes for which data were sought. Specify whether all results that were compatible with each outcome domain in each study were sought (e.g. for all measures, time points, analyses), and if not, the methods used to decide which results to collect.	Page 4, lines 166-171
	10b	List and define all other variables for which data were sought (e.g. participant and intervention characteristics, funding sources). Describe any assumptions made about any missing or unclear information.	Page 5, Lines 194-198
Study risk of bias assessment	11	Specify the methods used to assess risk of bias in the included studies, including details of the tool(s) used, how many reviewers assessed each study and whether they worked independently, and if applicable, details of automation tools used in the process.	Page 5, lines 200-213

Section and Topic	Item #	Checklist item	Location where item is reported
Effect measures	12	Specify for each outcome the effect measure(s) (e.g. risk ratio, mean difference) used in the synthesis or presentation of results.	Page 4, lines 183-184
Synthesis methods	13a	Describe the processes used to decide which studies were eligible for each synthesis (e.g. tabulating the study intervention characteristics and comparing against the planned groups for each synthesis (item #5)).	Page 4, lines 180-182
	13b	Describe any methods required to prepare the data for presentation or synthesis, such as handling of missing summary statistics, or data conversions.	Page 4, lines 182-183
	13c	Describe any methods used to tabulate or visually display results of individual studies and syntheses.	Page 4, line 179; Page 5, line 192
	13d	Describe any methods used to synthesize results and provide a rationale for the choice(s). If meta-analysis was performed, describe the model(s), method(s) to identify the presence and extent of statistical heterogeneity, and software package(s) used.	Page 4, line 184; Page 5, lines 191
	13e	Describe any methods used to explore possible causes of heterogeneity among study results (e.g. subgroup analysis, meta-regression).	Page 4, lines 186
	13f	Describe any sensitivity analyses conducted to assess robustness of the synthesized results.	Page 4-5, lines 186-187
Reporting bias assessment	14	Describe any methods used to assess risk of bias due to missing results in a synthesis (arising from reporting biases).	Page 5, line 204
Certainty assessment	15	Describe any methods used to assess certainty (or confidence) in the body of evidence for an outcome.	Page 4, line 184
**RESULTS**			
Study selection	16a	Describe the results of the search and selection process, from the number of records identified in the search to the number of studies included in the review, ideally using a flow diagram.	Page 6, line 235
	16b	Cite studies that might appear to meet the inclusion criteria, but which were excluded, and explain why they were excluded.	Supplemental Material
Study characteristics	17	Cite each included study and present its characteristics.	Supplemental Material
Risk of bias in studies	18	Present assessments of risk of bias for each included study.	Page 7, lines 253-254

Section and Topic	Item #	Checklist item	Location where item is reported
Results of individual studies	19	For all outcomes, present, for each study: (a) summary statistics for each group (where appropriate) and (b) an effect estimate and its precision (e.g. confidence/credible interval), ideally using structured tables or plots.	Page 7-11, lines
Results of syntheses	20a	For each synthesis, briefly summarise the characteristics and risk of bias among contributing studies.	Page 7-11, lines
	20b	Present results of all statistical syntheses conducted. If meta-analysis was done, present for each the summary estimate and its precision (e.g. confidence/credible interval) and measures of statistical heterogeneity. If comparing groups, describe the direction of the effect.	Page 7-11, lines lines 260-262, 276-280, 289, 300-301, 316, 325, 331, 338-339
	20c	Present results of all investigations of possible causes of heterogeneity among study results.	Page 7-11, lines lines 260-262, 276-280, 289, 300-301, 316, 325, 331
	20d	Present results of all sensitivity analyses conducted to assess the robustness of the synthesized results.	Page 7-11, lines 268-272, 281-284. 293-295, 307-310
Reporting biases	21	Present assessments of risk of bias due to missing results (arising from reporting biases) for each synthesis assessed.	Page 6, lines 242, 250
Certainty of evidence	22	Present assessments of certainty (or confidence) in the body of evidence for each outcome assessed.	Page 7-11, lines 260-262, 276-280, 289, 300-301, 316, 325, 331, 338-339
**DISCUSSION**			
Discussion	23a	Provide a general interpretation of the results in the context of other evidence.	Page 11, lines 346-350.

Section and Topic	Item #	Checklist item	Location where item is reported
	23b	Discuss any limitations of the evidence included in the review.	Page 12, lines 407 - 414
	23c	Discuss any limitations of the review processes used.	Page 12, lines 416-425
	23d	Discuss implications of the results for practice, policy, and future research.	Page 12, lines 400-405
**OTHER INFORMATION**			
Registration and protocol	24a	Provide registration information for the review, including register name and registration number, or state that the review was not registered.	Page 3, line 97-98
	24b	Indicate where the review protocol can be accessed, or state that a protocol was not prepared.	No
	24c	Describe and explain any amendments to information provided at registration or in the protocol.	N/A
Support	25	Describe sources of financial or non-financial support for the review, and the role of the funders or sponsors in the review.	Page 13, line 455
Competing interests	26	Declare any competing interests of review authors.	Page 13, line 458-459
Availability of data, code and other materials	27	Report which of the following are publicly available and where they can be found: template data collection forms; data extracted from included studies; data used for all analyses; analytic code; any other materials used in the review.	Supplemental materials
